# Gel-Based Materials for Intelligent Sensors and Self-Powered Nanogenerators

**DOI:** 10.3390/gels11110876

**Published:** 2025-11-01

**Authors:** Vineet Kumar, Sang-Shin Park

**Affiliations:** School of Mechanical Engineering, Yeungnam University, 280 Daehak-ro, Gyeongsan 38541, Gyeongbuk, Republic of Korea; vineetfri@gmail.com

## 1. Introduction

In recent years, gel-based sensors and self-powered nanogenerators have emerged as a promising class of novel materials with innovative applications, including wearable electronics, biomedical devices, and human–machine interfaces. These applications influence the unique properties of gel-based materials, particularly hydrogels and ionogels [1], which combine mechanical softness, high stretchability, biocompatibility, and tunable stiffness. Moreover, these materials can be tuned to balance electrical conductivity with energy harvesting and sensing functionalities. Gel-based sensors are soft, deformable devices that convert mechanical, thermal, or chemical stimuli into measurable electrical signals. They typically consist of a conductive gel matrix integrated with ionic or electronic fillers that enable signal transduction. Furthermore, they can undergo large deformations, easily conforming to irregular surfaces such as human skin [2]. Similarly, nanogenerators based on gels are designed to harvest external mechanical deformations or stimuli, such as ambient mechanical, thermal, or chemical energy, and convert them into electrical energy. Therefore, gel-based nanogenerators can eliminate the need for external batteries, especially in medical implants. They integrate piezoelectric, triboelectric, or ionic conduction mechanisms within flexible gel matrices [3]. Developing multi-responsive and self-healing gels for robust long-term operation is achievable by employing next-generation materials like MXene, MOFs, etc.

Considering these factors, this Special Issue focuses on the multifunctionalities of gel-based materials in terms of sensing and energy harvesting. For example, gel-based materials combine the flexibility and biocompatibility of soft gels for sensing systems [4], which use conductive hydrogels or ionogels to detect mechanical, thermal, or chemical stimuli using changes in their electrical properties. The softness, stretchability, and self-healing nature of these gel-based materials make them ideal for wearable and biomedical applications, as summarized in this Special Issue. Gel-based energy-harvesting systems that convert mechanical or environmental energy into electrical signals are also covered in this Special Issue. By integrating sensing and energy-harvesting capabilities, gel-based systems provide sustainable, flexible, and intelligent platforms [5] for wearable electronics, human–machine interfaces, and soft robotics. The ongoing research presented in this Special Issue focuses on improving the output performance and durability of these intelligent platforms. Finally, this Special Issue also focusses on next-generation smart, autonomous, and adaptive portable electronic sensors.

## 2. Overview of Published Articles

Periyasamy et al. [6] conducted a review of hydrogels regarding their use in transparent wearable technology. In this review, the authors describe the use of hydrogels in human–machine interactions, fostering health monitoring, advanced diagnostics, and augmented reality. They also discuss the future outlook regarding their use in wearable applications, as well as the critical challenges around their commercialization. Similarly, Singh et al. [7] conducted a critical study on gel-based nano-generators. This review describes the use of gel-based materials for next-generation smart applications, such as the Internet of Things, artificial intelligence, self-powered sensors, and portable electronics., This review will guide the robust design of high-performance sensors and their sustainable integration in next-generation wearable technologies. The research conducted by Garbel et al. [8] aimed to explore the fabrication of gels that could serve as a precursor to electrode material in supercapacitors. The presented asymmetric supercapacitor was based on a polyaniline matrix with xanthate-based embedded compounds. The results demonstrate that these supercapacitors exhibit robust performance, demonstrating high power density and durability, and therefore show potential for energy storage applications. Bottacin et al. [9] investigate nanocomposites based on silver-titana for photothermal applications. The results show that these composites are capable of local heating and can have heating effects up to tens of degrees. This work further demonstrates that these nanocomposites offer a promising pathway for the use of nano-systems in therapeutic applications.

In another study by Wang et al. [10], sensors were developed based on HEMA/AM/SA hydrogels. The results demonstrate that these fabricated sensors exhibit good stability, sensitivity, and selectivity. Finally, the fabricated sensors showed good potential for applications related to the monitoring of Pb^2+^ concentration in a particular subject. Similarly, in another study by Wang et al. [11], the authors developed hydrogels for electronic skin and information encryption. These hydrogels, developed using 3D printing, exhibited high conductivity, rapid UV response, and good color-changing reversibility. Due to the strong performance of these hydrogels, they are considered to show promise for use in various applications such as flexible strain sensors, information storage, and encryption devices. In a review by Sutradhar et al. [12], the authors reviewed the current prospects of TENGs. The main focus of this review was on conductivity and morphological engineering for sensing applications. The authors propose strategies to enhance energy harvesting in relation to triboelectric output and sensing sensitivity. Moreover, the key morphological features are reported, like surface roughness, porosity, and their impact on charge generation. Finally, this review critically examines a strategic roadmap for developing intelligent, sustainable, and multifunctional TENG-based sensing technologies. In the final paper, Li et al. [13] studied dielectric elastomer-based actuators for use in soft robotics. As the authors discuss, dielectric elastomer actuators (DEAs) have attracted significant attention because of their high energy density, rapid response, and excellent sensitivity. The authors developed and present the future prospects of these DEAs, along with their applications in medical and human–machine interfaces.

## 3. Summary and Future Outlook

These gel-based materials have novel prospects for energy and sensing engineering. By optimizing the unique mechanical and electrical tunability of these gels, we can create flexible, sustainable, and intelligent energy sensing platforms that can rapidly evolve the landscape of wearable and bio-integrated electronics [14]. Moreover, many gels exhibit reversible crosslinking, allowing for them to recover and heal after mechanical damage. Furthermore, some hydrogel-based sensors are water-rich and suitable for long-term contact with biological tissues. Therefore, their soft nature allows for even small external stimuli—such as changes in pressure, strain, humidity, or temperature—to induce measurable changes in resistance or capacitance. There are various types of gel-based sensors, including strain sensors, humidity sensors, pressure sensors, and biosensors [15], and these gel-based systems are paving the way toward intelligent, sustainable, and autonomous electronic devices, bridging the gap between soft materials and next-generation smart technologies.

Future studies on gel-based materials should focus on improving their novel aspects, such as energy efficiency, long-term stability, and high power density [16]. These aspects are important for interfacial engineering, encouraging hybrid systems, etc. Future developments should focus on advanced gel materials with enhanced mechanical resilience, conductivity, and self-healing abilities. Designs of hybrid gels that combine ionic liquids, conductive polymers, and nanomaterials will enable higher sensitivity, faster response, and improved durability. Next-generation nanomaterials include MXenes, graphene, carbon nanotubes, and boron nitride. The combination of gel-based systems with machine learning and deep learning algorithms will revolutionize their functionality [17]. AI can aid in pattern recognition, adaptive sensing, and real-time decision making. Moreover, future devices will increasingly focus on bio-integration because the water-rich and tissue-like nature of gels allows for their seamless contact with human skin or organs. Overall, these devices are expected to become the foundation for next-generation wearable healthcare, smart robotics, and autonomous electronic systems that will be useful in bridging the gap between living tissues and artificial intelligence.
